# Reliable Multi-Label Learning via Conformal Predictor and Random Forest for Syndrome Differentiation of Chronic Fatigue in Traditional Chinese Medicine

**DOI:** 10.1371/journal.pone.0099565

**Published:** 2014-06-11

**Authors:** Huazhen Wang, Xin Liu, Bing Lv, Fan Yang, Yanzhu Hong

**Affiliations:** 1 College of Computer Science and Technology, Huaqiao University, Xiamen, China; 2 School of Information Science and Engineering, Xiamen University, Xiamen, China; 3 Department of traditional Chinese medicine, Xiamen University, Xiamen, China; Taipei Medical University, Taiwan

## Abstract

**Objective:**

Chronic Fatigue (CF) still remains unclear about its etiology, pathophysiology, nomenclature and diagnostic criteria in the medical community. Traditional Chinese medicine (TCM) adopts a unique diagnostic method, namely ‘bian zheng lun zhi’ or syndrome differentiation, to diagnose the CF with a set of syndrome factors, which can be regarded as the Multi-Label Learning (MLL) problem in the machine learning literature. To obtain an effective and reliable diagnostic tool, we use Conformal Predictor (CP), Random Forest (RF) and Problem Transformation method (PT) for the syndrome differentiation of CF.

**Methods and Materials:**

In this work, using PT method, CP-RF is extended to handle MLL problem. CP-RF applies RF to measure the confidence level (p-value) of each label being the true label, and then selects multiple labels whose p-values are larger than the pre-defined significance level as the region prediction. In this paper, we compare the proposed CP-RF with typical CP-NBC(Naïve Bayes Classifier), CP-KNN(K-Nearest Neighbors) and ML-KNN on CF dataset, which consists of 736 cases. Specifically, 95 symptoms are used to identify CF, and four syndrome factors are employed in the syndrome differentiation, including ‘spleen deficiency’, ‘heart deficiency’, ‘liver stagnation’ and ‘qi deficiency’.

**The Results:**

CP-RF demonstrates an outstanding performance beyond CP-NBC, CP-KNN and ML-KNN under the general metrics of subset accuracy, hamming loss, one-error, coverage, ranking loss and average precision. Furthermore, the performance of CP-RF remains steady at the large scale of confidence levels from 80% to 100%, which indicates its robustness to the threshold determination. In addition, the confidence evaluation provided by CP is valid and well-calibrated.

**Conclusion:**

CP-RF not only offers outstanding performance but also provides valid confidence evaluation for the CF syndrome differentiation. It would be well applicable to TCM practitioners and facilitate the utilities of objective, effective and reliable computer-based diagnosis tool.

## Background

Chronic Fatigue (CF) is a sub-health status, pathologically characterized by nonspecific extreme fatigue (including physical fatigue and mental fatigue) over six months [1]. In the past, CF is a widespread illness which prevails among the people who lives under a fast-paced and stressful life. Thus far, the etiology, pathophysiology, nomenclature and diagnostic criteria of CF are still underexplored in Western medicine [2], [3]. Alternatively, Traditional Chinese Medicine (TCM) has provided an effective approach for personalized diagnosis and treatment of CF, and has paid increasing attention as a complementary medicine by the medical researchers [4], [5]. Unfortunately, TCM diagnosis still causes skepticism and criticism because TCM practitioners diagnose the patient only based on their subjective observation, knowledge, and clinical experience, which lacks objective test and cannot be scientifically proven by clinical trials [6]. Under the circumstances, it is desired to establish an objective and standardized diagnosis system for CF in TCM. Recently, researchers have found that machine learning technologies are able to figure out the inherent mechanism of TCM diagnosis and provide corrective predictions for patients [7], [8]. Therefore, a computer-aided system aiming at providing objective and reliable diagnosis is highly desired for the better understanding of the TCM diagnosis of chronic fatigue.

Differing from the western medicine, TCM adopts a unique diagnostic method, namely ‘*bian zheng lun zhi*’ or s*yndrome differentiation* [9]–[11], to practically diagnose the CF. According to the theory of TCM, the syndrome or *zheng* is a comprehensive description of the pathology of a disease in the body. Actually, the syndrome consists of a set of syndrome factors. Each factor is defined in terms of the *location* and *condition* of the body. The term *location* in TCM is similar to that of the Western medicine, such as heart, liver, spleen, lung, kidney and stomach. However, the term *condition* in TCM is totally different from the Western medicine, which reflects the disharmony in the body, such as the deficiency or excess of *qi*, *blood*, *yin* and *yang*. In the viewpoint of TCM, the body struggles to maintain a dynamic equilibrium between its internal conditions and the external environment. Several syndrome factors of the body will express simultaneously when a pathogenic disease occurs. In general, the total number of syndrome factors in TCM is about 60, and the related syndrome factors for a particular disease is a subset of all of the syndrome factors [12]–[14]. For example, the syndrome factors that applied for CF include ‘spleen deficiency’, ‘heart deficiency’, ‘liver stagnation’ and ‘*qi* deficiency’. When faced with a patient, TCM practitioners should first execute the manipulations of ‘inspection’, ‘auscultation-olfaction’, ‘interrogation’ and ‘palpation’ to identify the symptoms, and then diagnose which syndrome factors have expressed simultaneously and select them to be the diagnostic output for CF. Consequently, a corresponding TCM treatment is proposed based on these diagnosis results.

The above TCM diagnostic process can be seen as a pattern recognition process. The symptoms in TCM correspond to features in the machine learning literature, and syndrome factors serve as classes or labels. In this sense, the particular syndrome differentiation that diagnoses disease by a set of syndrome factors falls into the Multi-Label Learning (MLL) in the machine learning literature [15], [16]. Therefore, it is appropriate to design an MLL model for the diagnosis of chronic fatigue in TCM.

MLL technique addresses the learning setting where an instance is designated by a set of labels [17]–[19]. Accordingly, the MLL classifier would learn a discriminative function to output the region prediction (a set of labels) for the testing instance, which is different with the point prediction by traditional classifier. Further, the learning function can be regarded as the confidence measurement which measures the confidence of each label to be the true label or not. Given a pre-defined threshold, the irrelevant labels can be removed and the remaining ones are used to construct the region prediction [20]. Though MLL methods have been applied in various domains, such as image processing, text analysis and speech recognition, there are limited amount of research that applied to the syndrome differentiation in TCM. In the literature, a representative work is the application of ML-KNN (K-Nearest Neighbor) algorithm, which aims to diagnose the syndrome differentiation of coronary heart disease (based on 6 syndrome factors) [21], [22]. They computed the posterior probability of each label as the confidence measurement and selected those labels whose posterior probability is larger than a pre-defined threshold to construct the region prediction. The experiments have shown a promising performance for the syndrome differentiation.

Nevertheless, the reliability of the prediction result in the MLL framework has not been well studied, which is of crucial importance to the application of the high-risk medical diagnosis. That is, the reliable results are very important for the clinical treatment practically. As for the predictions of MLL model, it would be highly beneficial if the MLL model could provide a reliable analysis for the expert practitioners and patients. In general, the ML-KNN approach constructs the posterior probability as the confidence measurement for each label, provided that the proper prior assumption of the dataset distribution can be obtained [23]. However, it is impossible to properly figure out the prior knowledge about the dataset distribution because the TCM datasets are of high-dimensional and nonlinear patterns. Accordingly, the posterior probability always cannot provide valid confidence measurement for MLL prediction.

Motivated by the above observation, in this paper, we use Conformal Predictor (CP) and Random Forest (RF) to enhance the MLL framework, which aims to not only offer outstanding performance but also provide valid confidence for the syndrome differentiation of CF in TCM. CP is a recently development in the machine learning literature, which is virtually a confidence machine that tails its prediction with a valid confidence evaluation [24]. It has been proven that CP is calibrated in online learning, i.e. the accuracy of CP prediction can be hedged by the confidence level. In the past, CP has demonstrated the reliability on its prediction in many high-risk applications, such as medical diagnosis, fault detection and finance analysis [25]–[27]. CP outputs region prediction rather than point prediction, which makes it competent for the multi-label recognition. Meanwhile, RF is a powerful machine learning algorithm which can deal with dataset suffering heavily from high-dimensional, noisy, with missing-values, categorical and highly correlated features [28]. In this sense, RF is competent for TCM dataset where the descriptions of the symptom(features) always take categorical or qualitative values [29], [30]. Thus, CP and RF are suitable for modeling of syndrome differentiation of CF.

The method which combines CP and RF, namely CP-RF, was firstly proposed to deal with single-label classification problem in our previous work [36]. Unfortunately, CP-RF cannot be directly applied to syndrome differentiation of CF for it is a MLL problem. In this work, using Problem Transformation method, CP-RF is extended to handle MLL problem. **To the best of our knowledge, it is the first time that CP is applied to MLL tasks.**

The extended CP-RF was compared with two classical CP models CP-NBC (with Naive Bayes Classifier) and CP-KNN (with K-Nearest Neighbor) as well as the commonly used MLL algorithm in TCM, i.e. ML-KNN. Results of predictive effectiveness as well as some MLL-related evaluation metrics were reported. Especially, the validity of confidence measurement and the calibration property of CP-RF have been demonstrated. The experimental results show that CP-RF performs CP-NBC, CP-KNN and ML-KNN. In addition, the accuracy of CP-RF is higher than the confidence level, which reveals that the confidence evaluation of CP is valid and well-calibrated.

The remaining of this paper is organized as follows: in the Methods section the construction of the clinic CF dataset is introduced and the algorithmic process is proposed. In the Results section, the results of models constructed based on CP-RF, CP-NBC, CP-KNN and ML-KNN are compared. In the Discussion section, the reason that CP-RF can significantly improve the accuracy and provide valid confidence is discussed. The conclusive remarks followed in the Conclusion section.

## Methods

### Ethics Statement

N/A

### Dataset of Chronic Fatigue in TCM

In past years, we have done a substantial amount of work on the diagnosis of CF in TCM [31]. As for CF diagnosis in TCM, 80% of clinical identification were provided by ‘interrogation’ manipulation (inquiry) [21]. Therefore, the standardization inquiry system shall influence the diagnosis and treatment of CF significantly. In the past, we have designed a quantitative inquiry form to obtain the standardized inquiring items for the identification of CF. Accordingly, we have made an epidemiological investigation among a large number of people that come from the south of Fujian Province during August 2007 to December 2008. The participators were further constrained to the doctors, nurses and the teachers who worked in the colleges, the middle school and primary school. The participators who has a continuous or recurring fatigue over six months was accumulated into the CF dataset and each of them was further clinically identified by another three clinical manipulations (‘inspection’, ‘auscultation-olfaction’ and ‘palpation’). As shown in Table 1, 95 symptoms were finally collected to construct the complete symptom set (feature set) of CF.

**Table 1:** Set of symptom of CF in TCM.

ID	Symptoms				
1–5	depression	Fatigue afterexercise 24 hours	shortage of *qi*	pale complexion	sallow complexion
6–10	darkish complexion	bluish lip	gloomy complexion	fear of cold	fear of cold
11–15	vexing heat in thechest, palms and soles	afternoon fever	unsurfaced fever	tend to catch cold	spontaneous sweating
16–20	night sweating	pitting edema	cannot concentrate	amnesia	dim complexion
21–25	like sigh	thin	head stabbing pain	lassitude	heavy head
26–30	epilation or loose teeth	dry eyes	have a sudden blackoutwhen stand up	black eyes	tinnitus or deafness
31–35	dry throat	swollen pain inthe throat	discomfort in the throatlike something blockage	lymph node enlargement	lymph node tenderness
36–40	aching pain of neck	scurrying pain of theshoulder	stabbing pain of the waist	contracture of the back	oppression in the chest
41–45	palpitations	cough up thick phlegm	perennial cough	panting	stabbing pain in thechest or abdomen
46–50	distending and scurryingpain in the chest orabdomen	stuffiness andfullness in the chest	abdominal fullness	abdominal veins exposed	belching andacid vomiting
51–55	vomiting	abdominal distensionin the afternoonor after eating	numbness or paralysis	aching pain	distending pain
56–60	heavy body	encrusted skin	ache and weak in thewaist and kneeor heel pain	poor appetite	dry mouth
61–65	dry mouth and wantto drink	dry mouth butdon’t want to drink	bitter taste in the mouth	bland taste in the mouth	not thirst
66–70	insomnia	constipation	sloppy stool	sticky stool	stool sometimes sloppyand sometimes bound
71–75	reddish urine	yellow urine	frequent urination	copious and clear urine	dribbing urination
76–80	poor libido	dysmenorrhea	intermenstrual bleeding	menstrual irregularities	pale tongue
81–85	red tongue	enlarged tongue orteeth-marked tongue	spotted tongue	less fur	white and moist fur
86–90	yellow and slimy fur	string-like pulse	fine pulse	vacuous pulse	rough pulse
91–95	sunken pulse	relaxed pulse	slow pulse	rapid pulse	slippery pulse

According to the principle of syndrome differentiation in TCM, each symptom has a certain degree of influence on all of the syndrome factors. The contributions of each symptom to all of the syndrome factors differ from the others, which can be referenced from the lexicon of “Differentiation standards for symptoms and signs of Chronic Fatigue Disease in Traditional Chinese Medicine”. The expression level of a syndrome factor is determined by the fusion of all statistical frequencies of the related symptoms. In our previous study, we found that the frequently expressed syndrome factors of CF are ‘spleen deficiency’, ‘heart deficiency’ ‘liver depression’, ‘*qi* deficiency’ ‘*blood* deficiency’, ‘kidney deficiency’, ‘blood stasis’, ‘yang deficiency’, ‘lung deficiency’, and ‘phlegm turbid’. However, from a practical viewpoint, the former four syndrome factors are widely employed in the clinic diagnosis of CF and the others remained ambiguous effect to CF [31]. Accordingly, the most frequently occurring syndrome factors in the clinical practice, i.e., ‘spleen deficiency’, ‘heart deficiency’, ‘liver depression’ and ‘*qi* deficiency’ are employed to diagnose CF in TCM. As a result, 736 patients construct the CF dataset for our experiment. Each case is described by 95 symptoms (features) and a subset of the four syndrome factors (labels). The dataset is shown in Dataset S1 and data information is shown in Data information S1.

### Conformal Predictor

CP applies algorithmic randomness level associated with *p-value* scheme to measure the confidence of each label, and then selects the labels whose *p-values* are larger than a pre-defined significance level as the region prediction [32], [33]. A confidence level which is mutually complementary with the significance level is used to provide the confidence evaluation for the region prediction. The novelty of CP can be characterized by the calibration property of the region prediction, i.e. the accuracies of CP region prediction can be hedged by the confidence level. According to CP, given the training data sequence 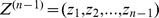 and the testing instance 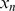, CP assumes all the possible labels 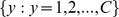 (*C* is the number of classes) being the candidate label for 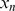, and then establish the corresponding testing example 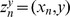. Thus, the testing data sequence is constructed by 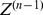 and each 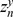. Consequently, there are *C* test data sequences, i.e
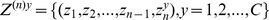
(1)


Secondly, CP applies algorithmic randomness statistical tests to test whether a particular testing data sequence 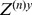 conforms to the independent and identical distribution (*i.i.d.)* or not. The algorithmic randomness level of 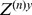 could be quantified by *p-value,* noted as 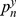. Intuitively, a small *p-value* means that 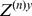
may not be an *i.i.d.* data sequence. It further implies that the corresponding candidate label 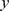 may not be the true label and should be discarded from the region prediction.

CP applies a unique method to construct the statistic *p-value*. CP designs a function 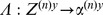, which maps each example 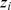 to a nonconformity score 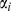, and thus establishes a one-dimension *nonconformity score sequence*:
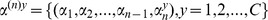
(2)where 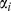 measures the degree of the nonconformity between 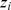 and 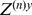. Based on 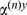, *p-value* is defined as follows:



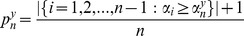
(3)In the end, the significance level ε,which reveals the smallest threshold of the acceptation of a particular testing data sequence 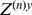 being the *i.i.d.* hypothesis, is used to be the threshold. Thus any testing data sequence 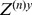 whose *p-values* are larger than the significance level should be the legal label and can serve as the true label. So CP outputs region prediction for 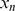 as follows,
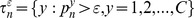
(4)


An error occurs when the prediction set 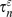 does not contain the true label 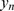 of the testing instance 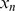. **It has been proven that in online learning setting the error rate of CP is not greater than significance level** 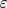, i.e.,
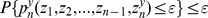
(5)


**Figure 1:**
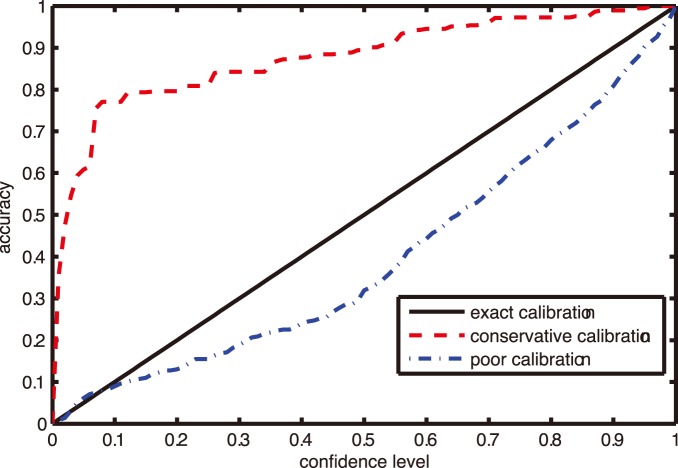
An illustrative example of calibration property.

The inequality (5) shows that the error rate of CP is bounded by the significance level. In the view of confidence level which is mutually complementary with the significance level, the accuracy of CP is hedged by the confidence level [34]. The relationship between accuracy and confidence level shows the calibration property of CP. Given different confidence levels, the performance of the corresponding CP accuracies is illustrated in Fig. 1. The abscissa represents the confidence level and the ordinate represents the corresponding accuracy of CP. In Fig. 1, the diagonal line with the legend of ‘exact calibration’ indicates that the accuracies of CP are equal to the corresponding confidence levels, and the accuracy rate curve hovering over exact calibration line denotes a conservative calibration property, while the curve lying below the diagonal line shows a poor calibration. In online setting, CP possesses exact or conservative calibration property in its region prediction, which enables it to provide valid confidence evaluation for its prediction.

According to CP, the computation of the nonconformity score 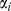 of 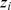 can be obtained generally by traditional machine learning algorithms, such as Support Vector Machine (SVM), K Nearest Neighbor (KNN) and Naïve Bayes classifier (NBC) [35]. CP-NBC plugs Naïve Bayes classifier (NBC) into the framework of CP which designs the posterior probability of label as the confidence measure, while CP-KNN designs the nonconformity score of example based on the Euclidean distances between the testing instance and its K nearest neighbor [35].

Considering the categorical characteristic of CF data which present a big challenge for the application of some distance metric-based algorithms, such as SVM and KNN, we plugged Random Forest (RF) into the framework of CP to construct CP-RF model for the syndrome differentiation of CF. RF is one of the most successful ensemble methods, which uses CART as its meta classifier [28]. RF repeats to draw bootstrap examples from the original dataset and then establishes *ntree* un-pruned CART trees. At each node of the CART tree, RF chooses randomly *mtry* features from the complete set of features to split. Based on the model of RF, a proximity measure between instances can be established. If instance 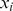 and 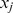 both land in the same terminal node of a CART tree, the proximity between them is increased by one, and the overall proximities, denoted as 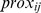 can be computed across all the CART trees. The nonconformity score based RF proximity is designed as follows:



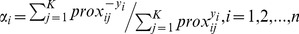
(6)where *K* is the number of nearest neighbors, 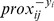
stands for the 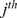 largest proximity between instance 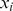 and the instances labelled differently from 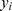; 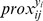
stands for the 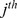 largest proximity between instance 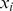
and the instances with the same label 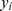. The intuition behind Equation (6) is that, two instances 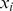 and 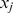 with the same label will tend to have a large proximity value and the two with different labels will have a small one. Thus the corresponding nonconformity score 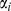 will be rather small, and vice versa. Therefore, the nonconformity measurement can exactly reflect the nonconformity of the example [36], [37].

### Multi-label Learning

In some pattern recognition tasks, the pattern of an instance can be described by multiple labels simultaneously. An image can be referred by multiple elements, such as mountain, lake and tree. The thematic topics of a text can include politic and education simultaneously [38]–[42]. Given each label 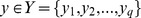 where *Y* denotes the label space with *q* possible class, the task of MLL is to learn a real valued function 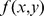 which measures the confidence of *y* being the proper label of *x*. Thus given a specified threshold 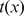, the MLL classifier should output the prediction 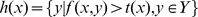, where 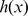 shows obviously to be a region prediction.

According to whether the multi-label examples would be transformed before modeling or not, the MLL algorithms can be divided into two categories: Problem Transformation methods (PT) and Algorithm Adaptation methods (AA) [17], [19]. PT method splits the multi-label examples straightforward into single-label examples and then applies single-label machine learning algorithms to tackle the multi-pattern recognition problem. Generally, six PT strategies have been reported in this issue. For example, the commonly used PT4 method transforms the original data set into *q* data sets. Each of them constructs a binary dataset which extracts the training instances relevant to a particular label as positive examples and the rest to be the negative examples. After applying the traditional machine learning algorithms to construct classifiers based on the *q* binary datasets, there must be a post-process mapping the traditional single-label outputs to multi-label prediction [43]. On the other hand, AA method adapts traditional single-label algorithms, such as KNN, SVM and boosting classifier to fit the multi-label data. The representative algorithms is ML-KNN [44]. For the testing instance *x*, the confidence score of each label is the posterior probability which is computed based on its K nearest distances and its conditional probability. Then the labels whose posterior probability are larger than a specific threshold (e.g., 0.5) should be selected as the prediction output.

### Using Conformal Predictor for Multi-label Learning

The main purpose of this work is to use CP method to construct an effective and reliable diagnostic tool for CF syndrome differentiation. CP outputs region prediction rather than point prediction, which makes it competent for the multi-label recognition task. However, the traditional machine learning algorithms which are plugged into the framework of CP to compute the nonconformity score of each example are always single-label machine learning algorithms. How to involve the multi-label examples into the framework of CP, i.e., how to measure the nonconformity score of each multi-label example and how to test the confidence level of a multi-label data sequence conforming to the *i.i.d.* assumption, has been the critical issue when using CP for multi-label learning.

**Figure 2:**
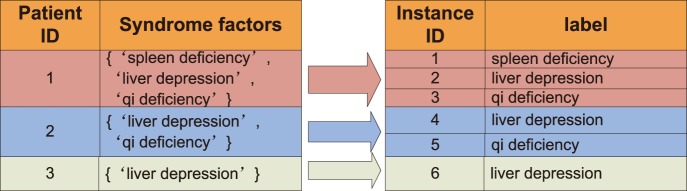
An illustrative example of the PT5 method.

In this study, in order to use CP in multi-label learning, we applied a simple and intuitive method, i.e., PT5 method [17]. Each multi-label instance *x* with a total *l* labels reproduces *l* single-label examples. As illustrated in Fig. 2, the patient (ID number 1) who has been diagnosed as (‘spleen deficiency’, ‘liver depression’, ‘*qi* deficiency’) will reproduce three instances with label ‘spleen deficiency’, ‘liver depression’ and deficiency’ respectively. With this method, the original multi-label CF dataset have transformed to a new single-label dataset which is suitable for single-label machine learning algorithms, such as NB, KNN and RF. Then CP-NBC, CP-KNN and CP-RF can be introduced to CF syndrome differentiation. When to predict a patient, CP-RF applies RF to measure the confidence level namely *p-value* of each label (syndrome factor) being the true label, and then selects multiple labels whose *p-values* are larger than the pre-defined significance level (threshold) as the region prediction. The confidence level which is mutually complementary with the significance level serves as the confidence evaluation for the region prediction.

Using PT5 method for data transformation and RF algorithm for nonconformity score computation, the algorithmic process of CP for multi-label learning is described as follows. The R code is shown in Code S1.

**Algorithm 1: CP-RF for Multi-Label Learning**

**Input:** 1. Training data sequence 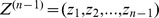 where each data 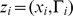 and 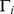 is the multi-label designation of instance 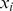.

2. A testing instance 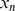

3. Significance level 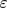

4. The parameters *ntree*, *mtry* of RF

5. The parameter *K* for nonconformity measurement

**Output:** region prediction 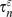

1. Applies problem transformation method(PT5) to get new training data sequence 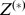

  1. Initiating 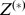 to be an empty set.
  2. for each data 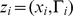

    - Measuring the size of 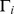 to be 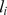

    - Reproducing the instance 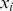 to the total of 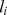 instances.
    - Designating each of the labels in 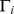 to the 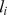 instance correspondingly.
    - Adding the new 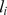 single-label examples into the transformed 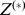.
2. Outputs the region prediction for instance 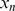 base on 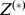 with the size of which being 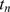

  1. Using 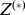 to construct RF model with the parameter *ntree*, *mtry*
  2. Exporting the proximity matrix 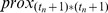 for the validating instance 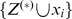.
  3. for 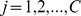 with 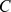 is the number of multiple labels
    - Applying equation (6) to obtain a serial of 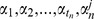 with the parameter *K*
    - Applying equation (3) to compute the algorithmic randomness level 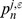

  4. Applying equation (4) to obtain the region output 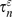

## Experimental Design and Evaluation

### Experiment Setup

The CP-RF model is compared with two classical CP models CP-NBC and CP-KNN as well as the commonly used ML-KNN in TCM. The detailed algorithm and parameter settings of CP-NBC and CP-KNN can be found in [35] and for MLL-KNN we refer to [21]. For CP-RF, the parameter *ntree* of RF is set to 1000 which is large enough, and *mtry* is 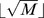 where *M* is the number of symptoms (features). The number of neighbors, *K*, which is required for CP-RF, CP-KNN and ML-KNN, we tried different values as *K* = 1, 3, 5, 7, 9,11.

All the algorithms were executed in leave-one-out cross-validation (LOOCV), which uses every single example of the original data set as the testing data, and the remaining examples as the training data in each fold. Compared with commonly used 5-fold and 10-fold cross-validation method, with LOOCV we can obtain more testing data to validate the statistical calibration property of the result on CF dataset.

### Evaluation Metric

Considering the particular multi-label learning setting, the evaluation metrics are different from metrics used in single-label learning. Given a pre-defined threshold 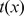, the MLL classifier will output the region prediction 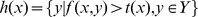. Let the result of label ranking for the testing instance 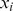 denoted as 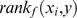, which is a one-to-one mapping onto 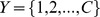 such that if 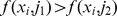
then 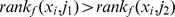 where 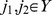. And the test dataset 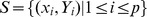, where 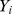 is the true label set for instance *x_i_* and *p* is the size of test dataset. Based on the above definition, the MLL-related evaluation metrics can be defined as follows [17], [19].

1. Subset Accuracy: The subset accuracy evaluates the accuracy of the multi - label classifier, which computes the fraction of the prediction region being identical to the true label set.
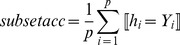
(7)
2. Hamming Loss: The hamming loss evaluates the fraction of misclassified instance-label pairs, i.e. a relevant label is missed or an irrelevant is predicted.
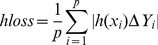
(8)where 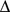 stands for the symmetric difference between two sets.
3. One-error: The one-error evaluates the fraction of examples whose top-ranked label is not in the true label set.
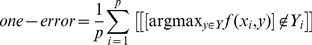
(9)
4. Coverage: The coverage evaluates how many steps are needed, on average, to move down the ranked label list so as to cover all the true labels of the example.
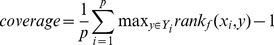
(10)
5. Ranking Loss: The ranking loss evaluates the fraction of reversely ordered label pairs, i.e., an irrelevant label is ranked higher than a relevant label.
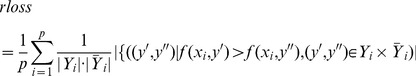
(11)
6. Average Precision: the average precision evaluates the average fraction of relevant labels ranked higher than a particular label actually being in the label set.

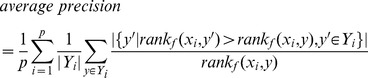
(12)According to the definition of the above six metrics, the higher the values of subset accuracy and average precision are preferred whereas the lower the values of hamming loss, one-error, coverage and ranking loss are welcome. Among them, *subset accuracy* and *hamming loss* evaluate the predictive effectiveness of the MLL model and are the most widely-used metrics in the MLL community.

### Verification of Reliability

As mentioned in Methods section, the most significant advantage of CP is the calibration property of its prediction, i.e., the error rate is exactly bounded by the predefined significance level. The calibrated prediction which provides valid confidence to evaluate the reliability of prediction is highly preferred to medical practitioners.

Theoretically, CP is well calibrated in the online setting. but extensive empirical studies have demonstrated that CP still shows good calibration property in batch learning, where the learning tasks are conducted by off-line learning [45], [46]. A typical example of this is the medical diagnosis, where only after a period of treatment can the prognostic information(true label) be obtained and thus the true label cannot be added timely for on-line learning. In this study we will empirically investigate the calibration property of CPs on CF dataset in LOOCV experiments.

Further, in previous studies, the calibration property of CP has only been tested on a single - label dataset. To the best of our knowledge, it is the first time that CP is applied to MLL tasks. Whether the calibration property still holds in MLL remains unknown in theory. Similar to single-label learning setting, we define the calibration property of MLL classifiers as follows: The risk of the true label set not being the subset of the prediction output is not greater then a specific significance level ε, i.e.,
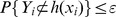
(13)


## Results

For a specified threshold, all the six evaluation metrics were computed based on the test data to evaluate the predictive performance of MLL classifiers. Given a series of different threshold values, the MLL classifiers output different prediction regions. For CP models, the threshold corresponds to a significance level while that of ML-KNN corresponds to posterior probability. In this sense, the preferred threshold values of CPs should be chosen from (0, 0.5) while the values of threshold of ML-KNN should be chosen from (0.5, 1). For the convenience of comparison, we use a confidence level (one minus significance level) as the threshold for CPs. Consequently, for all the algorithms, the higher the threshold is, the more reliable the prediction is. And the threshold values should range from 0.5 to 1, which is highly preferred in TCM practice.

### Comparison on Subset Accuracy and Hamming Loss

**Figure 3:**
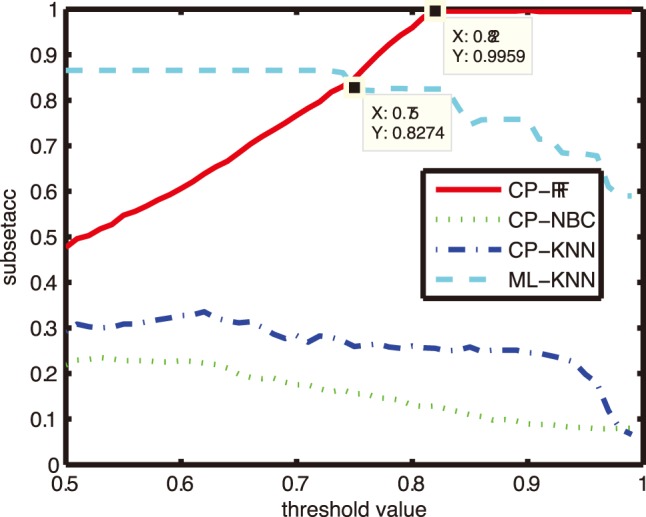
Comparison of subset accuracy with different thresholds.

**Figure 4:**
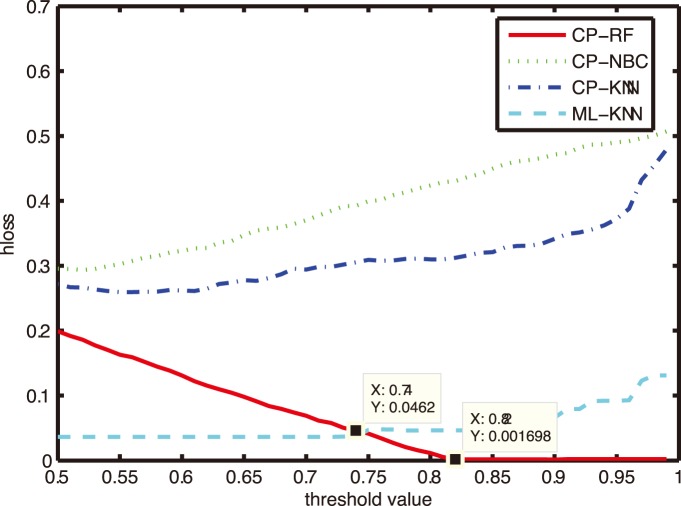
Comparison of hamming loss with different thresholds.

In this subsection, we compare the most commonly used metric - *subset accuracy* and *hamming loss*. The results of CP-RF, CP-NBC, CP-KNN and ML-KNN with parameter K = 1 were compared in Fig. 3–4, with the X axes representing the threshold value and the Y axes representing the subset accuracy and hamming loss respectively.

Fig. 3 illustrates the variation of subset accuracy with different threshold values from 0.5 to 1. From Fig. 3, we can see that the performances of CP-NBC,CP-KNN and ML-KNN deteriorate with the increase of the threshold, while the performance of CP-RF getting better. ML-KNN and CP-RF outperform CP-NBC and CP-KNN across the region (0.5, 1). CP-RF beats ML-KNN after the threshold value of 0.75 and CP-RF obtains the highest value 0.9959 of subset accuracy after the threshold value of 0.82.

The similar result can also be found from Fig. 4. The hamming loss of CP-NBC, CP-KNN and ML-KNN exhibit an increasing trend with the increase of a threshold value, while CP-RF gets lower hamming loss when the threshold value increases. Similarly, the performances of ML-KNN and CP-RF are significantly better than CP-NBC and CP-KNN across the region (0.5, 1). CP-RF outperforms ML-KNN after the threshold value of 0.75 and it obtains a lowest hamming loss value of 0.01698 after the threshold value of 0.82.

As discussed above, a higher threshold (higher confidence level or larger posterior probability) is preferred to medical practitioners. In this sense, CP-RF is a more effective and reliable classifier than ML-KNN and other CP models for CF diagnosis.

Further, in this study, CP-RF gets the same region prediction (high subset accuracy) at different thresholds ranging from about 0.8 to 1(high confidence level). In this sense, the size of prediction region, i.e., the number of syndrome factors selected by CP-RF model, is robust with the threshold determination to some context. The threshold determination remains an unsolved issue in MLL literatures [47]. The prior knowledge of the optimal threshold value has been always unavailable, except that a high confidence level is more preferred. As an example, we can see from Fig. 3 and Fig. 4, for ML-KNN, CP-NBC and CP-KNN, the performance always deteriorates with the increase of the threshold value. Researchers always have to trade-off between the reliability (higher threshold values) and effectiveness (performances) of the MLL classifiers. For example, Li et.al. set the threshold value to be 0.5 empirically [22], [48]. However, in this study, CP-RF does not suffer from this problem. Such merit provides marked significance for TCM syndrome differentiation.

### The Influence of Different *K* Values on Subset Accuracy and Hamming Loss

In order to investigate whether the *K* values will influence the performances of MLL classifiers, in this subsection we compare the 4 algorithms on subset accuracy and hamming loss with different *K* values. Results at three preferred confidence levels of 0.99, 0.9 and 0.8 were shown in Table 2 and Table 3. Limited by space, we only show the results with *K* = 5, 9 and 11.

**Table 2:** Comparisons of subset accuracy with different *K* values.

	*K* = 5			*K* = 9			*K* = 11		
**Confidence level**	0.99	0.90	0.80	0.99	0.90	0.80	0.99	0.90	0.80
**CP-RF**	0.94	0.97	0.98	0.93	0.97	0.99	0.93	0.97	0.99
**CP-NBC**	0.04	0.24	0.26	0.049	0.24	0.26	0.04	0.240	0.26
**CP-KNN**	0.10	0.33	0.38	0.10	0.31	0.37	0.10	0.34	0.40
**ML-KNN**	0.72	0.72	0.73	0.50	0.78	0.78	0.47	0.73	0.82

**Table 3:** Comparisons of hamming loss with different *K* values.

	*K* = 5			*K* = 9			*K* = 11		
**Confidence level**	0.99	0.90	0.80	0.99	0.90	0.80	0.99	0.90	0.80
**CP-RF**	0	0	0.01	0.02	0.01	0	0.02	0.901	0
**CP-NBC**	0.51	0.47	0.43	0.51	0.33	0.29	0.50	0.31	0.24
**CP-KNN**	0.51	0.35	0.31	0.50	0.35	0.31	0.51	0.35	0.31
**ML-KNN**	0.08	0.08	0.08	0.12	0.10	0.04	0.18	0.07	0.05

From Table 2, with different *K* values, CP-RF still performs the best compared with other algorithms at the specified confidence levels. The subset accuracy of all the four algorithms varies lightly with the variation of *K* values. Among them the fluctuations of CP-RF shows relatively small. Therefore, the performances of these algorithms are highly robust to the setting of *K*. The similar conclusion can be achieved from Table 3.

### Comparison on other Evaluation Metrics

**Figure 5:**
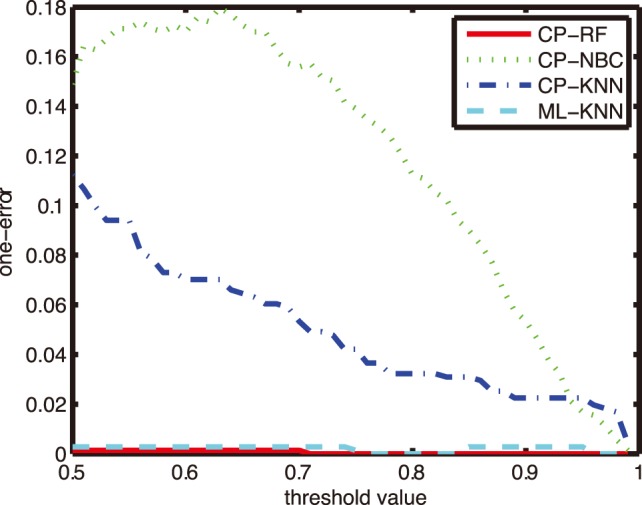
Comparison of one-error with different thresholds.

**Figure 6:**
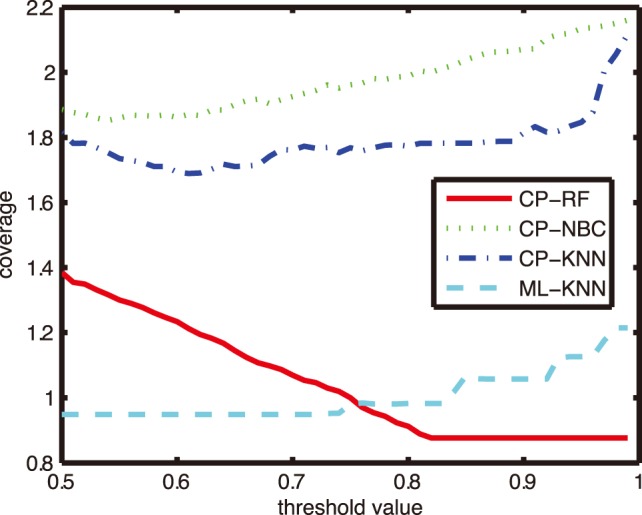
Comparison of coverage with different thresholds.

**Figure 7:**
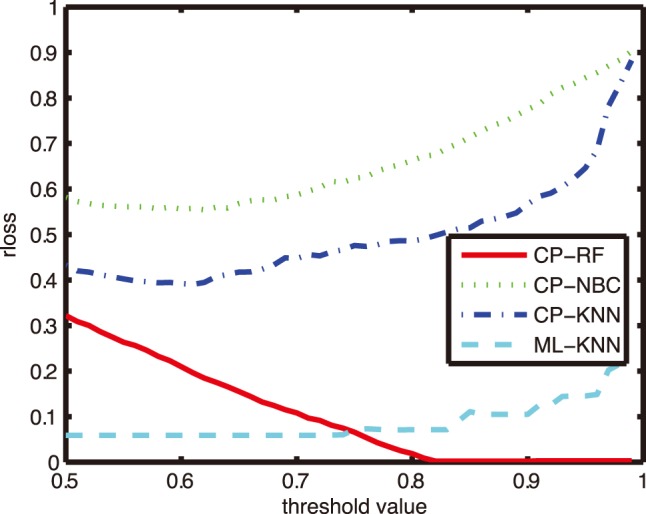
Comparison of ranking loss with different thresholds.

**Figure 8:**
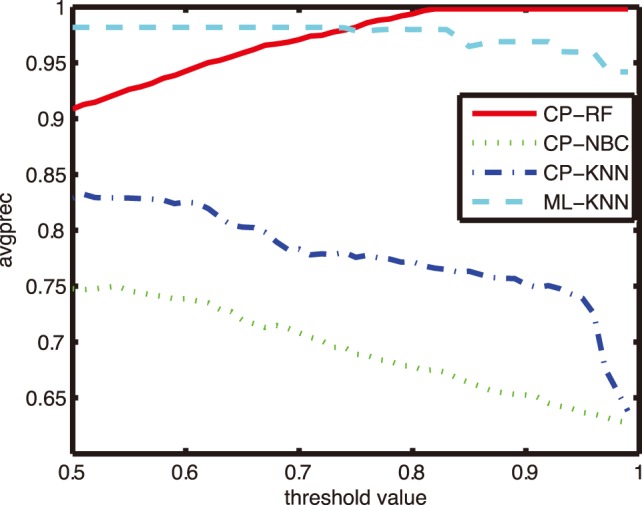
Comparison of average precision with different thresholds.

In order to further investigate the reliability and effectiveness of CP-RF models in the CF diagnosis, performances on other four evaluation metrics were reported. Fig. 5–8 shows the performance of four algorithms with parameter *K* = 1, and the comparative results with *K* = 5, 9 and 11 at three interested confidence levels of 0.99, 0.9 and 0.8 were listed in the Table 3–6.

**Table 4:** Results of one-error metric with different *K* values.

	*K* = 5			*K* = 9			*K* = 11		
**Confidence level**	0.99	0.90	0.80	0.99	0.90	0.80	0.99	0.90	0.80
**CP-RF**	0	0	0	0	0	0	0	0	0
**CP-NBC**	0.	0	0.01	0	0.01	0.06	0	0.01	0.01
**CP-KNN**	0	0.02	0.03	0	0.02	0.03	0.01	0.02	0.03
**ML-KNN**	0	0	0	0	0	0	0	0	0.03

**Table 5:** Results of coverage metric with different *K* values.

	*K* = 5			*K* = 9			*K* = 11		
**Confidence level**	0.99	0.90	0.80	0.99	0.90	0.80	0.99	0.90	0.80
**CP-RF**	0.90	0.88	0.88	0.90	0.89	0.88	0.91	0.89	0.88
**CP-NBC**	2.15	1.63	1.51	2.16	1.68	1.62	2.14	1.63	1.49
**CP-KNN**	2.20	1.83	1.78	2.17	1.83	1.78	2.19	1.83	1.78
**ML-KNN**	1.17	1.13	0.98	1.23	1.00	1.00	1.31	1.03	1.00

**Table 6:** Results of ranking loss metric with different *K* values.

	*K* = 5			*K* = 9			*K* = 11		
**Confidence level**	0.99	0.90	0.80	0.99	0.90	0.80	0.99	0.90	0.80
**CP-RF**	0.02	0.01	0.01	0.03	0.01	0.01	0.03	0.01	0.01
**CP-NBC**	0.90	0.51	0.43	0.90	0.56	0.50	0.89	0.50	0.40
**CP-KNN**	0.95	0.58	0.49	0.93	0.58	0.49	0.94	0.58	0.49
**ML-KNN**	0.23	0.20	0.08	0.29	0.11	0.09	0.34	0.14	0.09

As can be seen from Fig. 5–8, in case of three metrics, i.e., one error, coverage and ranking loss, which prefer small value, CP-RF achieve significantly small values which outperform the other three algorithms. Whereas in case of average precision metric which prefers high value, the CP-RF can get a significantly high value close to 1. The similar trend and result have been demonstrated in Table 4–7, regardless of the difference *K* values. The results in Fig. 5–8 and Table 4–7 indicate again that CP-RF would be a more effective and reliable tool for CF diagnosis.

**Table 7:** Results of average precision metric with different *K* values.

	*K* = 5			*K* = 9			*K* = 11		
**Confidence level**	0.99	0.90	0.80	0.99	0.90	0.80	0.99	0.90	0.80
**CP-RF**	0.99	0.99	1.00	0.99	0.99	1.00	0.98	0.99	1.00
**CP-NBC**	0.63	0.76	0.79	0.63	0.74	0.76	0.63	0.76	0.80
**CP-KNN**	0.62	0.75	0.77	0.63	0.75	0.77	0.62	0.75	0.77
**ML-KNN**	0.90	0.91	0.97	0.90	0.92	0.97	0.86	0.95	0.96

### The Calibration Property of CP Models

**Figure 9:**
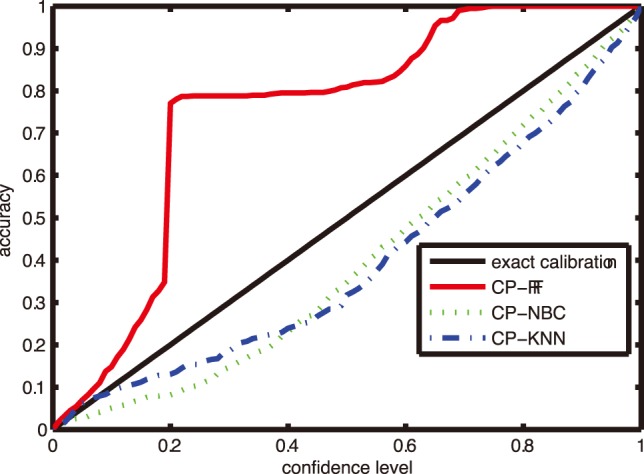
Calibration property of CPs on CF dataset.

In this subsection, we present a preliminary empirical investigation on the calibration property of CP on the CF dataset. Given different confidence levels, the corresponding accuracies were reported in Fig.9 for three CP models, i.e., CP-RF, CP-NBC and CP-KNN with *K* = 1. The X axis represents a series of different confidence levels and the Y axis strands for accuracy.

From Fig.9, we can see that the accuracy calibration line of CP-RF displays significantly beyond the exact calibration line, while the results of CP-KNN and CP-NBC show poor calibration. In this sense, CP-RF also outperforms CP-NBC and CP-KNN. The main reason may lie in the learning setting. Because the calibration property cannot be guaranteed theoretically in batch learning mode, in this case different nonconformity measurement with different algorithms (i.e. RF, KNN, and NBC) has a great impact on the performance of CP. The superiority of CP-RF will be explained in the Discussion section.

## Discussion

### Applicability of CP-RF Model for Chronic Fatigue Syndrome Differentiation

According to TCM theory, TCM diagnosis by the syndrome factor set is different from traditional single-pattern classification, and cannot be addressed by traditional single-label classifier. Attempts to solve the syndrome differentiation of CF have resulted in the development of multi-label learning, which can involve the complex interaction among different syndrome factors. Generally, the **syndrome differentiation of chronic fatigue** falls into multi-label classification setting [21], [49], [50].

Conformal Predictor (CP) can output region prediction tailed by valid confidence, which enables it to be a natural solution of multi-label learning. **However, using CP for multi-label learning has not yet been studied.** In this study, we applied it to chronic fatigue syndrome differentiation and verified its effectiveness and reliability.

Different CP models were used in this study, such as CP-NBC, CP-KNN and CP-RF. Among them, CP-RF which applies RF to compute the nonconformity score, achieved the best performance. The main reason may lie in the merits of the RF model when facing with CF dataset. RF highlights its superiority on the categorical CF dataset in TCM. The inferiority of CP-NBC may lie in the great dependency among the symptoms which deteriorates the performance of Naïve Bayes classifier, and the performances of CP-KNN and ML-KNN are affected by the categorical characteristic of the CF symptom.

The results also show that CP-RF remains steadily outstanding performance regardless of any threshold value among the region of (0.8, 1). Consequently, the size of prediction region, i.e., the number of syndrome factors selected by CP-RF model, is robust to the user-defined threshold, which remains an unsolved issue in many multi-label learning methods such as MLL-KNN [51], [52]. The robustness of CF syndrome differentiation by CP-RF has practical significance. Due to the substantially large amount of people suffer from CF, enormous health care resources tend to be consumed by patients with CF. However, different with acute illness that must be taken care of medical physician, CF treatments are mostly executed outside of the hospital. If the diagnosis of CF can be offered by the reliable computer-based intelligent tool using CP-RF, the patient can engage their own health more convenient and timely. In this sense, CP-RF can be employed as family-service equipment for the prevention and control for CF, which can dramatically relieve the burden of health care system.

Further, compared with ML-KNN, CP also offers valid confidence in the prediction region. The prediction region of CP is well-calibrated that the accuracy of region prediction is hedged by the specified confidence level.

### Contribution of CP-RF Model for Multi-label Learning

This study also indicated that CP can serve as a reliable MLL model. In details, the region prediction 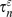 corresponds to 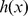, the algorithmic randomness level 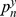 can be used as confidence scores for each possible label, and the significance level 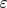 acts as the threshold determination. In this manner, the reliability or risk analysis of the prediction is emphasized, which is often neglected in MLL literatures.

Secondly, few studies have focused on the use of random forest in MLL. CP-RF which plugs RF into CP framework achieves superior performance on CF dataset and provides an alternative way of using random forest in MLL. In conclusion, CP-RF is a promising method for the MLL.

### Conclusions

Chronic fatigue syndrome differentiation has been formulated as a multi-label learning task. We plug random forest (RF) into the framework of conformal predictor (CP) to establish a reliable and effective diagnostic tool. Combined with PT5 method, CP-RF is extended to handle multi-label learning tasks. CP-RF outperforms CP-NBC, CP-KNN and MLL-KNN. Noted that in TCM medical diagnosis which is always conducted in batch learning mode, CP-RF still shows conservative calibration and provides valid confidence evaluation for the CF syndrome differentiation, which would be preferred to TCM practitioners.

## Supporting Information

Code S1 R code of CP-RF for Multi-Label Learning.
(R) Click here for additional data file.

Data information S1 Inclusion criteria of the patients, Diagnosis criteria, Exclusion criteria, Data Collection, Data Description of the Chronic Fatigue dataset used in this study.
(DOCX)

Dataset S1 The Chronic Fatigue dataset used in this study. 736 patients construct the CF dataset and each case is described by 95 symptoms (features) and a subset of the four syndrome factors (labels).
(TXT)
