# Supplementary material for: Reliable Multi-Label Learning via Conformal Predictor and Random Forest for Syndrome Differentiation of Chronic Fatigue in Traditional Chinese Medicine
Source: PLoS One. 2014 Jun 11;9(6):e99565. doi: 10.1371/journal.pone.0099565 (PMC4053362; doi:10.1371/journal.pone.0099565)
Supplement: Data information S1 — Inclusion criteria of the patients, Diagnosis criteria, Exclusion criteria, Data Collection, Data Description of the Chronic Fatigue dataset used in this study. (DOCX) [file pone.0099565.s002.docx]

## Data information

**Inclusion criteria of the patients**

1) The patients who meet the diagnostic criteria of CF; 2) The patients who are informed consented.

**Diagnosis criteria**

Diagnosis criteria of the patients are in western medicine and TCM. Diagnosis criteria in western medicine were established by the Centers for Disease Control and Prevention (CDC) of the U.S.A in 1994. CF is defined as a sub-health status, pathologically characterized by nonspecific extreme fatigue (including physical fatigue and mental fatigue) over six months. Diagnosis criteria in TCM are according to the “Differentiation standards for symptoms and signs of Chronic Fatigue in Traditional Chinese Medicine” in the " latest standard treatment for diseases " and " Differentiation standards for Deficiency Syndrome in Traditional Chinese Medicine", as well as the “Guideline for Clinical study of new drugs in Chinese herbs” and the lexicon of “Differentiation standards for symptoms and signs of Chronic Fatigue Disease in Traditional Chinese Medicine” in the textbooks.

**Exclusion criteria**

1) The Women in pregnancy or lactation; 2) The patients with mental diseases or with other severe diseases; 3) The patients who could not express their feeling clearly; 4) The patients who refused to participate in our study or without informed consent.

**Data Collection**

The patients with Chronic Fatigue disease are selected by the epidemiological investigation among a large number of people who come from the south of Fujian Province during August 2007 to December 2008. The participators were further constrained to the doctors, nurses and the teachers who worked in the colleges, the middle school and primary school. The participators who has a continuous or recurring fatigue over six months was accumulated into the CF dataset and each of them was further clinically identified by another three clinical manipulations (‘inspection’, ‘auscultation-olfaction’ and ‘palpation’). As a result, 736 patients construct the CF dataset for our experiment.

**Data Description**

Among the 736 patients, 567 patients are diagnosed with multi syndromes while only 169 patients are with single syndrome, regarding a total of four syndromes being used for the differentiation of CF in our study. According to TCM, each symptom has a certain degree of influence on all of the syndrome factors. The contributions of each symptom to all of the syndrome factors differ from the others. The expression level of a syndrome factor is determined by the fusion of all statistical frequencies of the related symptoms. In our previous study, we found that the frequently expressed syndrome factors of CF are ‘spleen deficiency’, ‘heart deficiency’ ‘liver depression’, ‘*qi* deficiency’ ‘*blood* deficiency’, ‘kidney deficiency’, ‘blood stasis’, ‘yang deficiency’, ‘lung deficiency’, and ‘phlegm turbid’. However, from a practical viewpoint, the former four syndrome factors are widely employed in the clinic diagnosis of CF and the others remained ambiguous effect to CF. After discussion with experts in cardiology, the most frequently occurring syndrome factors in the clinical practice, i.e., ‘spleen deficiency’, ‘heart deficiency’, ‘liver depression’ and ‘*qi* deficiency’ are employed to diagnose CF in TCM.
